# Altered Cortical Cholinergic Network in Parkinson’s Disease at Different Stage: A Resting-State fMRI Study

**DOI:** 10.3389/fnagi.2021.723948

**Published:** 2021-09-10

**Authors:** Wenshuang Sheng, Tao Guo, Cheng Zhou, Jingjing Wu, Ting Gao, Jiali Pu, Baorong Zhang, Minming Zhang, Yunjun Yang, Xiaojun Guan, Xiaojun Xu

**Affiliations:** ^1^Department of Radiology, The First Affiliated Hospital of Wenzhou Medical University, Wenzhou, China; ^2^Department of Radiology, The Second Affiliated Hospital, Zhejiang University School of Medicine, Hangzhou, China; ^3^Department of Neurology, The Second Affiliated Hospital, Zhejiang University School of Medicine, Hangzhou, China

**Keywords:** Parkinson’s disease, substantia innominata, cholinergic network, motor, functional connectivity

## Abstract

The cholinergic system is critical in Parkinson’s disease (PD) pathology, which accounts for various clinical symptoms in PD patients. The substantia innominata (SI) provides the main source of cortical cholinergic innervation. Previous studies revealed cholinergic-related dysfunction in PD pathology at early stage. Since PD is a progressive disorder, alterations of cholinergic system function along with the PD progression have yet to be elucidated. Seventy-nine PD patients, including thirty-five early-stage PD patients (PD-E) and forty-four middle-to-late stage PD patients (PD-M), and sixty-four healthy controls (HC) underwent brain magnetic resonance imaging and clinical assessments. We employed seed-based resting-state functional connectivity analysis to explore the cholinergic-related functional alterations. Correlation analysis was used to investigate the relationship between altered functional connectivity and the severity of motor symptoms in PD patients. Results showed that both PD-E and PD-M groups exhibited decreased functional connectivity between left SI and left frontal inferior opercularis areas and increased functional connectivity between left SI and left cingulum middle area as well as right primary motor and sensory areas when comparing with HC. At advanced stages of PD, functional connectivity in the right primary motor and sensory areas was further increased. These altered functional connectivity were also significantly correlated with the Unified Parkinson’s Disease Rating Scale motor scores. In conclusion, this study illustrated that altered cholinergic function plays an important role in the motor disruptions in PD patients both in early stage as well as during the progression of the disease.

## Introduction

Parkinson’s disease (PD), a chronic and progressive movement disorder, has been recognized as a heterogeneous syndrome (Zhang et al., 2005; Reich and Savitt, 2019). The pathological and neurotransmitter basis of PD is not all dopaminergic, other non-dopaminergic neurotransmitter systems are involved (Sethi, 2008), which leads to the heterogeneous clinical manifestations, not only including classic motor symptoms but involving differed extents of non-motor symptoms. Over the past decades, a major pathological emphasis has been placed on the disruption of dopaminergic system; however, it was found that the damage of non-dopaminergic system precedes the development of dopaminergic pathology (Braak et al., 2003), and has a profound influence on disease progression (Langston, 2006). Therefore, the investigation of the non-dopaminergic neurotransmitter system could provide a better understanding of the mechanism of PD.

Cholinergic system is a kind of major non-dopaminergic neurotransmitter system. Previous studies revealed that the abnormal function of cholinergic system is critical in the PD pathology (Mallet et al., 2019), which could account for various clinical symptoms in PD, including both motor symptoms (e.g., gait impairment, balance dysfunction and falls) (Bohnen et al., 2009; Bohnen and Albin, 2011; Lord et al., 2011; Yarnall et al., 2011; Rochester et al., 2012; Dalrymple et al., 2021) and non-motor symptoms (e.g., cognition impairment and visual hallucinations) (Yarnall et al., 2011; Kim et al., 2017; Lee et al., 2018). The substantia innominata (SI) in the basal forebrain is the major sources of cholinergic projections in the brain (Mesulam and Geula, 1988; Li et al., 2014). The loss of SI neurons represents cortical cholinergic deficits (Perry et al., 1985). According to the classic PD pathology model, basal forebrain is a main target of the α-synuclein accumulation (Braak et al., 2003), and the α-synuclein accumulation in which area simultaneously occurs with the accumulation in the substantia nigra at the early stage of PD (Lee et al., 2018). A positron emission tomography (PET) study of cerebral acetylcholinesterase demonstrated that cholinergic dysfunction occurs in the early course of PD (Hughes et al., 1992; Latt et al., 2009). This evidence suggests that the pathology of early PD involves the cholinergic-related dysfunction. Given that PD is a progressive disorder, patients at advanced stages seem to have a significantly faster disease progression (Zhao et al., 2010) and show a high incidence of balance dysfunction (Latt et al., 2009). These heterogeneous clinical symptoms indicate that the degenerative mechanisms of the cholinergic system may be different during PD progression, which have yet to be elucidated.

Advanced magnetic resonance imaging (MRI) technology provides an avenue to explore the cholinergic function in PD patients. Currently, MRI studies have found a piece of evidence indicating the abnormal cholinergic function in PD patients. A whole brain voxel-based morphometry study has found that the gray matter density of SI was reduced in PD patients and it was associated with gait impairment (specially reduced gait speed) as well as balance dysfunction (Dalrymple et al., 2021). Other studies using resting-state functional MRI (rs-fMRI) revealed that in PD patients, altered cholinergic network of SI was significantly correlated with cognitive performance (Kim et al., 2017; Lee et al., 2018). However, these studies mainly focused on the specific PD population, such as PD patients at early stage or patients before the surgical stage. Researchers argue that functional alterations likely precede structural atrophy and examination of cerebral functional connectivity may be essential to understanding the etiologies of many neuropsychiatric disease (Liang et al., 2011; Guan et al., 2019). Low-frequency fluctuations of resting-state blood oxygenation level-dependent (BOLD) signal reflect connectivity between functionally related brain regions (Fox and Raichle, 2007). So resting-state functional connectivity (rsFC) can be used to evaluate altered relationships between the SI and particular areas of the whole brain thus defining brain regions related to the severity of motor symptoms. Earlier studies of rsFC have mainly focused on the role of cholinergic function in cognition (Kim et al., 2017; Lee et al., 2018). Taken together, considering the progressive characteristics of PD as we mentioned above, exploring the cholinergic function of PD patients at different stages would help us understand the pathophysiological mechanism with PD progression better.

On the basis of a previous study (George et al., 2011), we firstly segmented bilateral SI in the individual high-resolution structural images and defined them as seeds in the following rsFC calculation. We aimed to explore the altered cholinergic function in different stages of PD patients via the measure of functional connectivity of SI and investigate the relationships between aberrant SI connectivity and the disease severity. We hypothesized that with the progression of disease, PD patients’ SI-FC would be disrupted and associated with the disease severity.

## Materials and Methods

### Participants

All participants were recruited from the Department of Neurology, Second Affiliated Hospital of Zhejiang University and this study was approved by the Medical Ethics Committee of The Second Affiliated Hospital of Zhejiang University School of Medicine and the ethical approval number was (2017) Ethical Approval Study No. 008. Written informed consent was obtained from all participants before enrollment in the study. We excluded participants with a history of anticholinergic drugs, a history of neurologic or psychiatric disorders, brain trauma, or general exclusion criteria for MR scanning and analyzing. Specifically, seven normal controls and eight patients with PD were excluded for the following reasons: (1) with significant motion artifact during scanning, *n* = 6; (2) with severe brain atrophy or enlarged ventricles, *n* = 4; (3) with poor coregistration results, *n* = 2; (4) with incomplete demographic information, *n* = 3. After exclusion, 79 patients with PD and 64 healthy controls (HC) were included in this study. PD was diagnosed by a senior neurologist (BZ) according to the United Kingdom PD Society Brain Bank criteria (Hughes et al., 1992). For PD patients who were under antiparkinsonian treatment, MRI scanning and clinical assessments were performed in the morning after withdrawing all antiparkinsonian drugs overnight (at least 12 h) (on “drug-off status”). Basic demographic information, including age, gender, education duration, drug state and neurologic, and psychiatric scales including Hoehn-Yahr stage, Unified Parkinson’s Disease Rating Scale (UPDRS) and Montreal Cognitive Assessment (MoCA) score were obtained from all PD patients. UPDRS motor scores were divided into subscores of axial symptoms (items 27–30). According to Hoehn-Yahr stage, PD patients were divided into two groups: 35 patients with Hoehn-Yahr stage ≤ 1.5 were grouped into early-stage PD group (PD-E) and 44 patients with Hoehn-Yahr stage ≥ 2 were grouped into middle-to-late stage PD group (PD-M) (Li et al., 2020). For HC, basic demographic information and MoCA score were recorded.

### MRI Data Acquisition

All participants were scanned on a 3.0-Tesla MRI scanner (GE Discovery 750) equipped with an 8-channel head coil. During MRI scanning, the head was stabilized using restraining foam pads, and earplugs were provided to reduce the noise during scanning. Structural T1 images were acquired using a fast spoiled gradient recalled sequence: repetition time = 7.336 ms; echo time = 3.036 ms; inversion time = 450 ms; flip angle = 11°; field of view = 260 × 260 mm^2^; matrix = 256 × 256; slice thickness = 1.2 mm; 196 continuous sagittal slices. Rs-fMRI images were acquired using gradient recalled echo-echo planar imaging sequence: repetition time = 2,000 ms; echo time = 30 ms; flip angle = 77°; field of view = 240 × 240 mm^2^; matrix = 64 × 64; slice thickness = 4 mm; slice gap = 0 mm; 38 interleaved axial slices. During MRI scanning, subjects were instructed to remain awake with their eyes closed and not to move or focus on a specific thought.

### Seed Definition and Normalization

**FIGURE 1:**
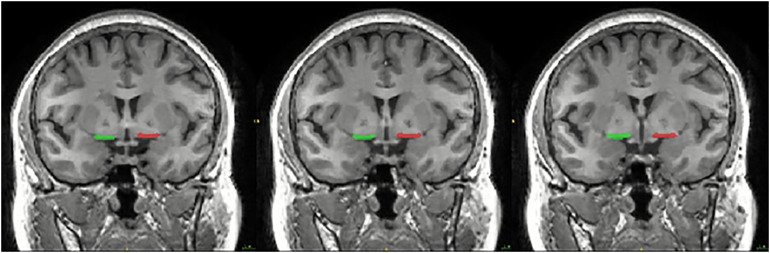
Coronal slice of the MRI image showing the location of the substantia innominate (SI) used in the seed-based resting-state functional connectivity (rsFC) analysis. The SI was drawn at three consecutive gapless 1.2 mm-thick slices on T1-weighted coronal images reformatted to be perpendicular to the anterior commissure (AC)-posterior commissure (PC) line. The three consecutive sections analyzed were located at the level of the crossing of the AC, the level where the AC might be uncrossed, and the level of the emergence of the AC from the temporal lobe. The boundaries of the SI were as follows: the dorsal border was the ventral aspect of the globus pallidus, the ventral border was the base of the brain containing the anterior perforated space, the medial border was operationally defined by a vertical line extending from the ventrolateral border of the bed nucleus of the stria terminalis to the base of the brain, and the lateral border extended to the medial aspect of the putamen.

The bilateral SI were manually drawn on the coronal T1-weighted MRI images by a radiologist who was blinded to the participants’ identity according to the method provided by George et al. (2011). Specifically, the SI was drawn at three consecutive gapless 1.2 mm-thick slices on T1-weighted coronal images reformatted to be perpendicular to the anterior commissure (AC)-posterior commissure (PC) line. The three consecutive sections analyzed were located at the level of the crossing of the AC, the level where the AC might be uncrossed, and the level of the emergence of the AC from the temporal lobe. The boundaries of the SI were as follows: the dorsal border was the ventral aspect of the globus pallidus, the ventral border was the base of the brain containing the anterior perforated space, the medial border was operationally defined by a vertical line extending from the ventrolateral border of the bed nucleus of the stria terminalis to the base of the brain, and the lateral border extended to the medial aspect of the putamen. The SI delineation of each section was shown in Figure 1.

The bilateral SI normalization was conducted using the VBM8 toolbox^1^ implemented in SPM8.^2^ All native T1-weighted images were normalized to the standard Montreal Neurological Institute (MNI) space. The corresponding normalization parameters were then applied to the bilateral SI, and therefore native SI was transformed into standard MNI space. After the SI normalization, a group-based probability map of bilateral SI was generated, and a threshold of 0.4 was used to obtain the binary SI mask (de Flores et al., 2017).

### MRI Data Preprocessing and Functional Connectivity Analysis

The rs-fMRI data preprocessing was performed using the standard pipeline in the Data Processing and Analysis for (Resting-State) Brain Imaging suite (see text footnote 2) (Yan et al., 2016), which included first 10 volumes removing, slice timing, realignment, the nuisance covariates regression (Friston 24 head motion parameters, white matter, and cerebrospinal fluid signal), spatial normalization through structure images, smoothing with a gaussian kernel of 6 × 6 × 6 mm^3^ full width at half maximum, temporal band-pass filtering (0.01–0.1 Hz), linear detrending, and scrubbing. No subject showed apparent head motion over 2 mm (transformation) and/or 2°(rotation). The bilateral SI mask in MNI space was used as seed, respectively, and a seed-based functional connectivity in whole brain was performed. Fisher’s r-to-z transformation was applied to improve data’s normality for parametric statistical analysis.

### Statistical Analysis

Statistical analyses of demographic and clinical data were performed using SPSS 20.0 statistical software. Categorical variables were assessed using chi-square tests. The one-sample Kolmogorov-Smirnov test was used to check the data’s normality. Normally distributed continuous variables were assessed using independent sample *t* test or analysis of variance (ANOVA). Non-parametric data were assessed using Wilcoxon rank-sum test and Kruskal-Wallis test. LSD correction was performed for the multiple comparisons in *post hoc* analyses. Two-tailed *p* < 0.05 was regarded as significant.

To locate the brain regions with significant difference in functional connectivity with SI among the three groups, one-way analysis of covariance (ANCOVA) was performed by using the statistical analysis tool of DPABI (Yan et al., 2016). Age, sex, and education duration were incorporated as covariates. Gaussian Random Field (GRF) correction (voxel *p* = 0.001, cluster *p* = 0.05, two-tailed) was used for multivoxel comparisons. Then, the mean FC of each individual in these significantly differed regions was extracted to perform the group comparisons as well as *post hoc* analyses. Least-Significant Difference (LSD) correction was used to correct the multiple comparisons in *post hoc* analyses. Two-tailed *p* < 0.05 was regarded as significant.

To test whether functional connectivity in brain regions showing significant group difference was correlated with the disease severity indicated by motor symptoms, partial correlation analysis was then conducted to evaluate the relationship between the FC and raw scores of UPDRS III, controlling age, gender and education duration as covariates. Bonferroni correction was performed for the multiple comparisons in *post hoc* analyses. Statistical significance was set at *p* < 0.05.

## Results

### Demographic and Clinical Characteristics

Demographic and clinical characteristics of all participants were shown in Table 1. No significant differences in gender (*p* = 0.140), education duration (*p* = 0.359) and MoCA score (*p* = 0.555) were observed between PD-E, PD-M and HC. No significant differences in disease duration (*p* = 0.275) and drug state (*p* = 0.813) were observed between PD-E and PD-M. And the details of drug state of each PD patient were shown in Supplementary Table 1, none of them have a history of anticholinergic drugs. The age (*p* = 0.027) of PD-M is older than PD-E. Hoehn-Yahr stage (*p* < 0.001) and UPDRS III scores (*p* < 0.001) of PD-M were significantly higher than PD-E.

**TABLE 1:** Demographic and clinical information for the participants.

	**PD-E (*n* = 35)**	**PD-M (*n* = 44)**	**HC (*n* = 64)**	***P* value**	***Post hoc* analysis**		
					**PD-E vs PD-M**	**PD-E vs HC**	**PD-M vs HC**
Age, years	58.64 (6.66)	60.38 (6.60)	59.49 (8.25)	0.072	0.027	0.070	0.538
Sex, F/M	16/19	18/26	38/26	0.140	–	–	–
Education, years	9.25 (4.02)	8.84 (3.24)	9.78 (3.15)	0.359	0.591	0.459	0.158
Disease duration, years	3.59 (3.71)	4.59 (4.24)	–	–	0.275	–	–
Drug-naïve, yes/no	6/29	8/36	–	–	0.813	–	–
H-Y	1.10 (0.20)	2.35 (0.45)	–	–	0	–	–
UPDRS-III	14.20 (5.00)	28.32 (12.21)	–	–	0	–	–
Axial symptoms	2.37 (1.21)	3.82 (1.96)	–	–	0	–	–
MoCA	24.28 (3.57)	23.48 (4.32)	24.13 (3.15)	0.555	0.329	0.834	0.366

*H-Y, Hoehn-Yahr stage; UPDRS-III, part III of the Unified Parkinson’s Disease Rating Scale; MoCA, Montreal Cognitive Assessment.*

### Comparative Analysis of Functional Connectivity Between SI and the Rest of Brain Regions

**FIGURE 2:**
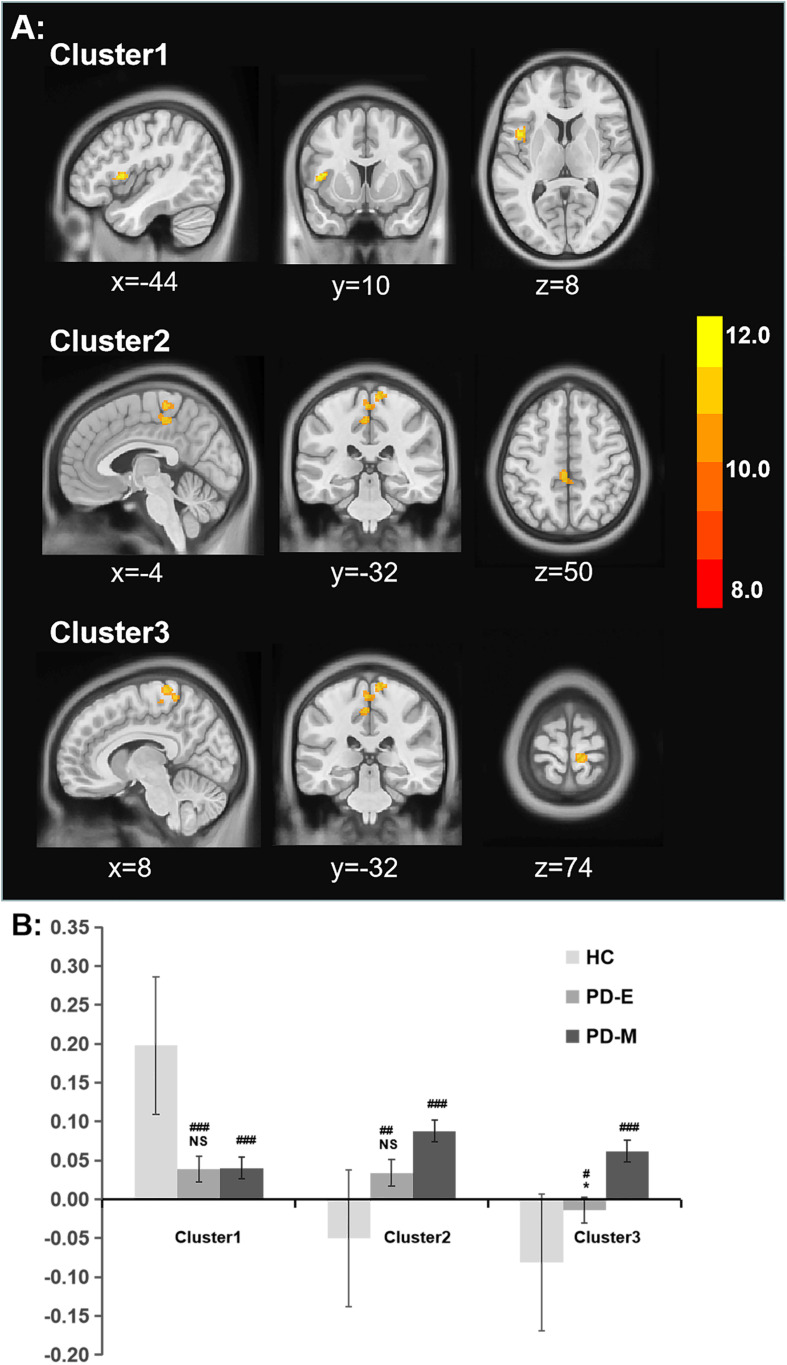
**(A)** Significant alterations of rsFC between the left SI and whole brain among all groups. **(B)** The bar plot of comparisons of functional connectivity between groups. *p < 0.05 vs the PD-M group, ^#^*p* < 0.05; ^#^^#^*p* < 0.01; and ^#^^#^^#^*p* < 0.001 vs the HC group. rsFC, resting-state functional connectivity; SI, substantia innominate.

Brain regions showing significant difference of functional connectivity between left SI and the rest of brain among three groups were shown in Figure 2A, and the comparisons of functional connectivity between groups were shown in Figure 2B. Anatomical location and *post hoc* comparison results of altered functional connectivity in significant brain regions were shown in Table 2. Compared with HC, both PD-E and PD-M groups showed decreased functional connectivity in left frontal inferior opercularis areas, partly extending to the left insula (cluster 1), and increased functional connectivity in the left cingulum middle area (cluster 2) and right primary motor and sensory areas (cluster 3). Interestingly, functional connectivity in the right primary motor and sensory areas (cluster 3) continued to increase in PD-M group when compared with PD-E group. These results indicated that even in the early stages of PD, cortical cholinergic denervation has occurred in left frontal inferior opercularis areas (cluster 1), and cholinergic hyperactivity has occurred in the left cingulum middle area (cluster 2) and right primary motor and sensory areas (cluster 3). And at advanced stages of PD, cholinergic hyperactivity in the right primary motor and sensory areas would develop further. There was no difference of functional connectivity between right SI and the rest of brain among three groups.

**TABLE 2:** Anatomical location and *post hoc* comparison results of altered functional connectivity in significant brain regions.

**Brain region**	**Number of voxels**	**Side**	**Peak MNI coordinate**			**F**	**The Mean FC of Each Cluster**			***Post hoc* analysis**			**MD**		
			**X**	**Y**	**Z**		**PD-E**	**PD-M**	**HC**	**PD-E vs PD-M**	**PD-E vs HC**	**PD-M vs HC**	**PD-E vs PD-M**	**PD-E vs HC**	**PD-M vs HC**
Cluster 1	98		–44	10	8	14.12	0.039 (0.127)	0.040 (0.202)	0.198 (0.183)	0.979	<0.001	<0.001	–0.011	–0.159	–0.158
Frontal Inferior Opercularis	66	L													
Insula	27	L													
Cluster 2	91		–4	–32	50	11.45	0.034 (0.133)	0.088 (0.162)	–0.050 (0.151)	0.114	0.009	<0.001	–0.054	0.084	0.138
Paracentral Lobule	47	L													
Cingulum Middle	40	L													
Cluster 3	317		8	–32	74	13.27	–0.014 (0.153)	0.062 (0.148)	–0.081 (0.130)	0.02	0.026	<0.001	–0.076	0.067	0.142
Paracentral Lobule	142	R													
Paracentral Lobule	70	L													
Precentral gyrus	24	R													
Postcentral gyrus	12	R													

*FC, functional connectivity; MD, mean difference; L, left; R, right.*

### Correlation Analysis of UPDRS III Scores and Resting State Functional Connectivity

**FIGURE 3:**
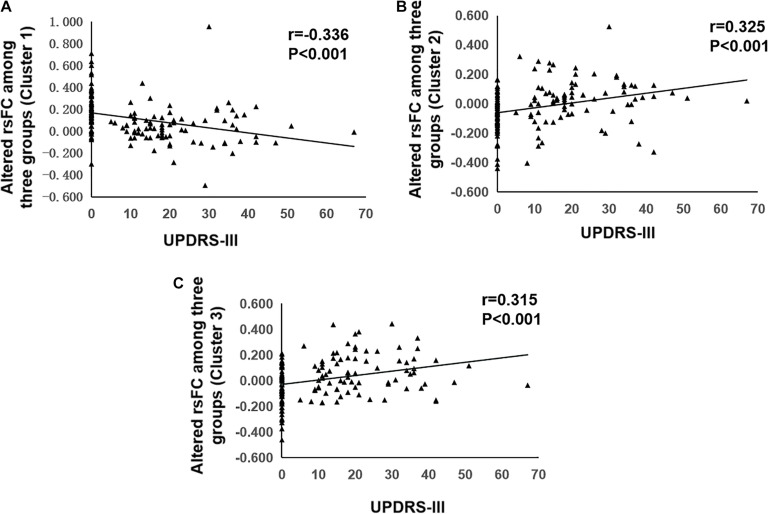
Correlation analysis of UPDRS-III and resting state functional connectivity. **(A)** Correlation analysis of UPDRS-III and altered rsFC of Cluster 1. **(B)** Correlation analysis of UPDRS-III and altered rsFC of Cluster 2. **(C)** Correlation analysis of UPDRS-III and altered rsFC of Cluster 3. rsFC, resting-state functional connectivity; UPDRS-III, part III of the Unified Parkinson’s Disease Rating Scale.

The results of correlation analyses were shown in Figure 3. Partial correlation analysis showed that decreased functional connectivity in left frontal inferior opercularis and insula area (cluster 1) was negatively correlated with UPDRS III scores (*r* = –0.336, *p* < 0.001) and subscores of axial symptoms (*r* = –0.261, *p* = 0.002). Increased functional connectivity in left cingulum middle area (cluster 2) and right primary motor and sensory areas (cluster 3) was positively correlated with UPDRS III scores (*r* = 0.315, *p* < 0.001, and *r* = 0.325, *p* < 0.001, respectively) and subscores of axial symptoms (*r* = 0.342, *p* < 0.001, and *r* = 0.355, *p* < 0.001, respectively).

## Discussion

The main findings of the present study were as follows: (1) The decreased functional connectivity in left frontal inferior opercularis areas, partly extending to the left insula (cluster 1), and increased functional connectivity in the left cingulum middle area (cluster 2) and right primary motor and sensory areas (cluster 3) were both shown in PD-E and PD-M groups when compared with HC; (2) at advanced stages of PD, functional connectivity in the right primary motor and sensory areas (cluster 3) was further increased; (3) decreased functional connectivity in left frontal inferior opercularis and insula area (cluster 1) was negatively correlated with UPDRS III scores and subscores of axial symptoms. And increased functional connectivity in left cingulum middle area (cluster 2) and right primary motor and sensory areas (cluster 3) was positively correlated with UPDRS III scores and subscores of axial symptoms.

Compared with the HC, we found that PD patients showed decreased functional connectivity between left SI and left frontal inferior opercularis, indicating the cholinergic denervation in the frontal inferior opercularis. A PET study found the neuroinflammation of SI in patients with REM sleep behavior disorder, which would lead to cortical cholinergic dysfunction in the frontal inferior opercularis (Staer et al., 2020). Frontal inferior opercularis plays a key role in postural and gait control, and an impairment of this area might lead to abnormal postural and gait control. Previous studies showed that the disruption of the projection from the SI to frontal inferior opercularis may result to the deficiency in the information processing from the temporoparietal cortex to the frontal cortex, which may cause errors in anticipatory postural adjustment and gait difficulties (Takakusaki, 2017; Vastik et al., 2017). And a MRI study showed that the decreased cortical thickness in frontal inferior opercularis was associated with motor symptoms (e.g., gait impairment) in PD (Herman et al., 2014; Vastik et al., 2017). This evidence indicated that the disrupted function of frontal inferior opercularis driving by the cholinergic degeneration was related to the motor difficulties. In this study, we found that the cortical cholinergic denervation in left frontal inferior opercularis was negatively associated with UPDRS motor scores and subscores of axial symptoms, which further supported that the cholinergic dysfunction in frontal inferior opercularis was associated with the severity of motor symptoms in PD patients.

In this study, we found increased functional connectivity between SI and cingulum middle areas both in PD-E and PD-M patients, which indicated the cholinergic hyperactivity in cingulum middle areas in PD patients. The cingulate gyrus is an important component of the limbic system which has rich distribution and intensity of acetylcholinesterase containing fiber (Mesulam et al., 1984). Previous study found the functional connectivity increases in the cingulate gyrus in PD patients with mild cognitive impairment (Zhan et al., 2018), which was in line with our findings. Former studies revealed that the generation and release of acetylcholine (ACh) and dopamine (DA) are both reduced in PD, however, overall acetylcholine was in a dominant position, resulting to a relatively cholinergic hyperactivity; further, the preponderance of ACh over DA contributes to the motor deficit (McKinley et al., 2019). An animal study showed that the parkinsonian motor dysfunction could be relieved by locally injecting the botulinum neurotoxin A in order to reduce the release of ACh (Wree et al., 2011). These studies indicated that cholinergic hyperactivity was related to the motor deficits in PD. In this study, we found the cholinergic functional connectivity in cingulum middle areas was positively correlated with UPDRS III scores and subscores of axial symptoms, suggesting that the more cholinergic hyperconnectivity in cingulum middle areas, the more severe of motor symptoms, which were similar to the previous studies. Taken together, we proposed that cholinergic hyperactivity in cingulum middle area may cause severe motor symptoms in PD patients.

An interesting finding in this study was the progressively increment of the cholinergic functional connectivity in primary motor and sensory areas at advanced stages of PD. A previous PET study found that specific populations (e.g., patients with movement disorders) exhibited decreased acetylcholinesterase activity in paracentral lobule, precentral gyrus, and postcentral gyrus (Hirano et al., 2010), pointing to the increased activity of cholinergic function in these brain regions. Intriguingly, some studies found the functional connectivity in primary motor and sensory areas was increased (Onu et al., 2015) and a greater improvement in UPDRS-III scores following L-dopa administration was characterized by lower functional connectivity in primary motor and sensory area, which were in agreement with our findings (Akram et al., 2017). Additionally, we found that the hyperactivity in primary motor and sensory areas was positively correlated with UPDRS III score and subscores of axial symptoms, suggesting a disease severity relevant role of cholinergic hyperactivity of these regions. Considering the progressive characteristic of PD evolution, we supposed that the cholinergic hyperconnectivity in primary motor and sensory areas may be a crucial mechanism for the disease progression.

There are some limitations of this study. First, selecting seed of seed-based rsFC analysis must be based on previous literature, which is subjective and cannot fully explore altered functional connectivity of the whole brain. Second, the sample size of this study was moderate. Third, in this study, we did not find any correlation between general cognition function (MoCa) and cholinergic network. Because most of the patients did not have multiple-domain cognition assessment, current finding should be cautiously translated to other relevant studies. And future studies are warranted to further disclose this interesting topic. Finally, this study is retrospective and cross-sectional. Further prospective and longitudinal studies with a larger sample size are expected to validate these finds and, importantly, to explore the longitudinal alterations of cholinergic-related functional connectivity along the disease progression, which could provide greater insight into the cholinergic neuromechanism of PD progression.

In conclusion, this study revealed altered cholinergic functional connectivity in PD patients, which were associated with the severity of motor symptoms. Specifically, cholinergic functional connectivity in primary motor and sensory cortex was progressively increased at advanced stages of PD. These findings illustrated that altered cholinergic function plays an important role in the motor disruptions in PD patients both in early stage as well as during the progression of the disease.

## Data Availability Statement

The original contributions presented in the study are included in the article/Supplementary Material, further inquiries can be directed to the corresponding authors.

## Ethics Statement

The studies involving human participants were reviewed and approved by the Medical Ethics Committee of The Second Affiliated Hospital of Zhejiang University School of Medicine. The patients/participants provided their written informed consent to participate in this study.

## Author Contributions

XX, XG, YY, and MZ were responsible for the study concept and design. WS wrote the main manuscript text. TGu revised the main manuscript text. WS, TGa, CZ, JW, TGu, JP, and BZ contributed to the acquisition of imaging data. WS, TGu, CZ, and JW performed data analysis and interpreted the findings. All authors contributed to the article and approved the submitted version.

## Conflict of Interest

The authors declare that the research was conducted in the absence of any commercial or financial relationships that could be construed as a potential conflict of interest.

## Publisher’s Note

All claims expressed in this article are solely those of the authors and do not necessarily represent those of their affiliated organizations, or those of the publisher, the editors and the reviewers. Any product that may be evaluated in this article, or claim that may be made by its manufacturer, is not guaranteed or endorsed by the publisher.

## Footnotes

1
http://dbm.neuro.uni-jena.de/vbm

2
http://www.fil.ion.ucl.ac.uk/spm/
