# Supplementary material for: Altered Cortical Cholinergic Network in Parkinson’s Disease at Different Stage: A Resting-State fMRI Study
Source: Front Aging Neurosci. 2021 Sep 10;13:723948. doi: 10.3389/fnagi.2021.723948 (PMC8461333; doi:10.3389/fnagi.2021.723948)
Supplement: Supplementary file 1 [file Table_1.docx]

**Table S1** The details of medication state of PD patients

| **Number** | **drug** | **Group** | **Levodopa and BenseraZide Hydrochloride Tablets** | **Amantadine Tablets** | **Carzodopa sustained-release tablets** | **Pramipexole Dihydrochloride Tablets** | **Selegiline Hydrochloride Tablets** | **Piribedil sustained-release tablets** | **Entacapone Tablets** | **Selegiline Hydrochloride Tablets** |
| --- | --- | --- | --- | --- | --- | --- | --- | --- | --- | --- |
| PD001 | 1 | 1 | 1.25# | 2# |  |  |  |  |  |  |
| PD002 | 1 | 1 |  |  | 1# | 6# |  |  |  |  |
| PD003 | 1 | 1 | 1.5# |  |  |  | 1# |  |  |  |
| PD004 | 1 | 1 | 1.5# | 2# |  |  |  |  |  |  |
| PD005 | 1 | 1 | 1.5# |  |  |  | 1# |  |  |  |
| PD006 | 1 | 1 | 0.75# |  |  | 1.5# |  |  |  |  |
| PD007 | 0 | 1 |  |  |  |  |  |  |  |  |
| PD008 | 1 | 1 | 1.5# |  |  |  |  |  |  |  |
| PD009 | 1 | 1 | 1# |  |  |  | 2# |  |  |  |
| PD010 | 1 | 1 | 1.5# |  |  |  |  |  |  |  |
| PD011 | 1 | 1 |  |  |  | 1.5# |  | 3# |  |  |
| PD012 | 1 | 1 | 0.75# |  |  |  |  |  |  | 2# |
| PD013 | 1 | 1 | 0.75# |  |  |  |  |  |  | 2# |
| PD014 | 1 | 1 | 1.5# |  |  |  |  |  |  |  |
| PD015 | 1 | 1 | 1.5# |  |  | 3# |  |  |  |  |
| PD016 | 0 | 1 |  |  |  |  |  |  |  |  |
| PD017 | 1 | 1 | 0.75# |  |  | 6# |  |  |  |  |
| PD018 | 1 | 1 |  | 2# | 1# | 9# |  |  |  |  |
| PD019 | 1 | 1 |  |  | 1# |  | 2# |  |  |  |
| PD020 | 0 | 1 |  |  |  |  |  |  |  |  |
| PD021 | 1 | 1 | 1.5# |  |  |  | 2# |  |  |  |
| PD022 | 1 | 1 | 2# |  |  | 6# | 2# |  |  |  |
| PD023 | 1 | 1 | 1.5# | 2# |  |  |  |  |  |  |
| PD024 | 1 | 1 | 2# |  |  | 1.5# |  |  |  |  |
| PD025 | 1 | 1 | 1.5# |  |  | 3# |  |  |  |  |
| PD026 | 1 | 1 |  |  |  | 3# |  |  |  |  |
| PD027 | 1 | 1 | 1.5# |  |  |  |  |  |  |  |
| PD028 | 0 | 1 |  |  |  |  |  |  |  |  |
| PD029 | 1 | 1 | 1.5# |  |  | 3# |  |  |  |  |
| PD030 | 1 | 1 |  |  |  |  | 2# |  |  |  |
| PD031 | 1 | 1 | 1# |  |  |  |  |  |  |  |
| PD032 | 1 | 1 | 2# |  |  |  |  |  |  |  |
| PD033 | 0 | 1 |  |  |  |  |  |  |  |  |
| PD034 | 0 | 1 |  |  |  |  |  |  |  |  |
| PD035 | 1 | 1 | 0.25# |  |  |  |  | 3# |  |  |
| PD036 | 1 | 2 | 1.5# |  |  |  |  | 3# |  | 2# |
| PD037 | 1 | 2 |  | 1# | 1.5# | 3# | 1# |  |  |  |
| PD038 | 1 | 2 | 1.5# |  |  |  |  |  |  |  |
| PD039 | 0 | 2 |  |  |  |  |  |  |  |  |
| PD040 | 0 | 2 |  |  |  |  |  |  |  |  |
| PD041 | 1 | 2 | 1.5# |  |  |  | 2# |  |  |  |
| PD042 | 1 | 2 | 1.5# | 2# | 2# |  |  | 1# | 1# |  |
| PD043 | 0 | 2 |  |  |  |  |  |  |  |  |
| PD044 | 1 | 2 | 2.5# | 2# |  |  |  |  |  |  |
| PD045 | 1 | 2 | 1.5# | 2# | 0.5# |  |  |  |  |  |
| PD046 | 1 | 2 | 1.5# |  |  |  |  |  |  |  |
| PD047 | 1 | 2 | 2# |  |  | 2# |  |  |  |  |
| PD048 | 1 | 2 |  |  | 3# |  |  |  |  | 1# |
| PD049 | 1 | 2 | 2# |  |  |  |  |  |  | 2# |
| PD050 | 1 | 2 | 3# | 1# |  | 3# |  |  |  |  |
| PD051 | 1 | 2 | 2# |  |  | 6# |  |  |  | 2# |
| PD052 | 1 | 2 | 1.5# |  |  | 6# |  |  |  |  |
| PD053 | 1 | 2 |  |  | 1# |  | 1# |  |  |  |
| PD054 | 1 | 2 | 1# | 2# |  |  |  |  |  |  |
| PD055 | 1 | 2 | 1.5# |  |  | 6# |  |  |  |  |
| PD056 | 1 | 2 | 3# |  |  | 6# |  |  | 1.5# |  |
| PD057 | 1 | 2 | 4# | 2# |  |  |  |  |  | 2# |
| PD058 | 1 | 2 | 0.75# |  |  |  |  |  |  | 1# |
| PD059 | 1 | 2 | 1.5# | 2# |  |  |  |  |  |  |
| PD060 | 1 | 2 | 1.5# |  |  |  |  |  |  | 2# |
| PD061 | 1 | 2 | 2# |  |  |  |  |  |  | 1# |
| PD062 | 0 | 2 |  |  |  |  |  |  |  |  |
| PD063 | 1 | 2 | 3# |  |  | 3# |  |  |  |  |
| PD064 | 1 | 2 | 1.5# |  |  |  |  |  |  |  |
| PD065 | 1 | 2 | 0.75# |  |  | 3# |  |  |  |  |
| PD066 | 0 | 2 |  |  |  |  |  |  |  |  |
| PD067 | 1 | 2 | 1# |  |  |  |  |  |  |  |
| PD068 | 0 | 2 |  |  |  |  |  |  |  |  |
| PD069 | 1 | 2 | 2# |  |  | 2# |  |  |  |  |
| PD070 | 1 | 2 | 1.5# |  |  |  |  |  |  | 1# |
| PD071 | 1 | 2 | 1.5# | 2# |  | 3# |  |  |  |  |
| PD072 | 1 | 2 | 2# | 2# |  |  | 2# |  | 1.5# |  |
| PD073 | 1 | 2 | 0.75# |  |  |  |  | 2# |  |  |
| PD074 | 1 | 2 | 1.5# | 3# |  | 6# |  |  |  |  |
| PD075 | 1 | 2 | 2# |  |  |  |  | 1# |  |  |
| PD076 | 1 | 2 | 0.75# |  |  |  |  |  |  | 2# |
| PD077 | 1 | 2 | 0.5# |  |  | 3# |  |  |  |  |
| PD078 | 0 | 2 |  |  |  |  |  |  |  |  |
| PD079 | 0 | 2 |  |  |  |  |  |  |  |  |

#: A pill
